# Therapeutic Insights and Immune Pathway Connections Revealed by Core Symptom Gene Network Analysis in Ankylosing Spondylitis

**DOI:** 10.3390/cimb48020199

**Published:** 2026-02-11

**Authors:** La Yoon Choi, Mi Hye Kim, Dae Yong Kim

**Affiliations:** College of Korean Medicine, Woosuk University, 61, Seonneomeo 3-gil, Wansan-gu, Jeonju-si 54986, Jeonbuk-do, Republic of Korea

**Keywords:** ankylosing spondylitis, network pharmacology, symptoms, molecular heterogeneity, pathway enrichment

## Abstract

Ankylosing spondylitis (AS) exhibits marked clinical heterogeneity that is poorly captured by conventional disease-centric analyses, hindering the development of personalized therapies. We propose a symptom-centered network pharmacology framework that directly links individual clinical symptoms to their underlying molecular mechanisms and therapeutic targets. AS- and symptom-associated genes were collected from GeneCards and prioritized using centrality analysis within protein–protein interaction networks. Symptom relevance was validated using patient-derived transcriptomic datasets. Network proximity between symptom modules and FDA-approved drug targets was assessed. A refined gene set, integrating *TNF*-associated neighbors and highly central nodes, was subjected to pathway enrichment analysis. Disease-centric analysis yielded a restricted 18-gene core enriched mainly in broad immune pathways. In contrast, the symptom-centered network identified 145 genes associated with specific symptoms such as inflammatory back pain and morning stiffness. Key genes, including *PTEN*, *TLR4*, *JAK2*, *NRAS*, and *NR3C1*, were significantly upregulated in AS patients. *TNF* showed local connectivity but limited global proximity, while *IL17A*- and *JAK* inhibitor-related targets were absent. A refined 24-gene module revealed enrichment in interleukin- and cytokine-mediated signaling pathways. Symptom-centered network analysis more effectively captures molecular heterogeneity in AS, providing a robust framework for symptom-specific target discovery and personalized therapeutic strategies.

## 1. Introduction

Ankylosing spondylitis (AS) is a chronic inflammatory rheumatic disease occurring due to a form of spondyloarthritis (SpA) that primarily affects the axial skeleton, particularly the spine and sacroiliac joints [1]. Patients typically experience back pain, morning stiffness, and progressive restriction of spinal mobility, which can ultimately lead to severe postural deformities and radiographic ankylosis, a condition commonly known as the “bamboo spine” [2,3]. These debilitating musculoskeletal changes significantly impair physical function, reduce work capacity, and diminish a patient’s overall quality of life severely [4]. Furthermore, AS is frequently accompanied by extra-articular manifestations, including acute anterior uveitis, inflammatory bowel disease (IBD), psoriasis, and cardiovascular comorbidities, further complicating disease management [5].

The pathogenesis of AS is a complex, multifactorial process involving a dynamic interplay of genetic predisposition, environmental exposures, and immune dysregulation [6]. The human leukocyte antigen-B27 (HLA-B27) gene is strongly associated with the disease in approximately 80–95% of patients. Nonetheless, only a fraction of HLA-B27-positive individuals develop AS, indicating the involvement of other genetic variants, such as endoplasmic reticulum aminopeptidase 1 (*ERAP1*), interleukin-23 receptor (*IL23R*), and tumor necrosis factor superfamily member 15 (*TNFSF15*) [7,8]. Environmental triggers, particularly disruptions in the gut microbiota and bacterial antigens, are thought to contribute through molecular mimicry with HLA-B27 [9]. Additionally, multiple factors such as smoking, vitamin D deficiency, and biomechanical stress at the entheses have been linked to disease activity and progression [10]. AS is primarily an autoimmune disease with the misfolding of HLA-B27 heavy chains can induce endoplasmic reticulum stress and trigger the unfolded protein response. Similarly, the IL-23/Th17 axis drives an excessive production of IL-17, TNF-α, and other cytokines, leading to chronic inflammation and abnormal bone remodeling characteristic of AS [11,12]. This persistent inflammation at the entheses and axial skeleton eventually triggers both bone resorption and new bone formation, ultimately causing spinal fusion and irreversible structural damage [13].

All treatments for AS focus on alleviating pain and stiffness, maintaining mobility, and preventing complications at the moment. There are non-steroidal anti-inflammatory drugs (NSAIDs), TNF inhibitors, IL-17A inhibitors, and janus kinase (JAK) inhibitors for AS patients [1]. Despite the development of therapeutic agents that target these key pathways, treatment outcomes remain suboptimal [14]. NSAIDs remain the first-line treatment, effectively alleviating pain and stiffness [15]. However, their long-term use is limited by the risk of gastrointestinal, cardiovascular, and renal toxicity [16]. For patients who respond inadequately to NSAIDs, biologic agents targeting TNF have demonstrated significant efficacy in reducing inflammation and delaying structural progression [16]. TNF inhibitors (etanercept, adalimumab, and golimumab), IL-17A inhibitors (secukinumab and ixekizumab), and JAK inhibitors (upadacitinib and tofacitinib) have expanded the therapeutic landscape for AS [17]. Nonetheless, these agents are associated with adverse events, including increased susceptibility to infections, injection-site reactions, and paradoxical inflammatory responses [18,19], with up to 30% of patients experiencing primary or secondary non-responsiveness [20]. TNF inhibitors exert their biological properties by blocking TNF-α activity and thereby reducing joint inflammation [19]. IL-17 inhibitors provide an additional therapeutic option for patients who do not respond adequately to TNF inhibitors [21]. Moreover, the patients who fail to NSAIDs, TNF inhibitors or IL-17A inhibitors, JAK inhibitors serve as alternative treatment options [17]. However, these treatments also carry risks, including upper respiratory tract infections, the exacerbation of inflammatory bowel disease, and long-term risk of malignancies and cardiovascular events [22,23]. The significant variability in treatment response underscores the critical need for personalized medicine, a necessity stemming from the complexity and diversity of AS pathogenesis. This therapeutic variability highlights that current limitations are closely linked to our incomplete understanding of the underlying molecular networks. Given that many complex diseases, including AS, arise from perturbations across interconnected pathways rather than isolated genetic defects, the conventional ‘one drug–one target’ paradigm is insufficient [24]. A broader systems-level framework is therefore essential to capture the multifactorial nature of AS and to identify clinically relevant points of intervention [25,26].

Network pharmacology has emerged as a promising framework to address these challenges by integrating multi-omics data, protein–protein interaction networks, and pathway analyses to identify key nodes and interactions underlying disease processes [27]. This approach enables the simultaneous examination of multiple targets and pathways, offering the potential to elucidate disease mechanisms, predict drug responses, and guide the design of multi-target therapeutic strategies [28]. In the context of AS, network pharmacology provides a unique opportunity to explore the interplay between symptom-associated genes and existing drug targets, thereby uncovering new intervention points and refining current treatment approaches [29]. Nonetheless, disease-centric network analyses, which dominate prior studies, tend to emphasize broad canonical hubs while overlooking the molecular diversity that underlies distinct clinical manifestations. However, most previous network-based investigations of AS have concentrated on disease-level gene sets. This approach obscures the molecular diversity driving distinct clinical manifestations such as inflammatory back pain, morning stiffness, or peripheral arthritis. This gap limits the translational relevance of studies containing therapeutic strategies derived from purely disease-centric analyses, which may fail to capture the symptom-specific mechanisms that directly impact patient quality of life. To address this limitation, we shift the paradigm from a traditional disease-centric model to a novel symptom-centered network pharmacology framework. This study is, to our knowledge, one of the first to computationally dissect the molecular heterogeneity of AS based on its core symptoms. We systematically identified core genes associated with major AS symptoms, validated their expression patterns in patient datasets, and examined their topological relationships with TNF, a principal therapeutic target in AS management. By combining centrality-based network analysis, gene expression validation, and network proximity assessment, we aimed to generate novel insights into the systems-level mechanisms of AS and to inform the development of more precise and effective therapeutic strategies.

## 2. Materials and Methods

### 2.1. Gene Set Construction

FDA-approved therapeutic targets for AS were identified through DrugBank (https://go.drugbank.com/, accessed on 5 September 2025). To construct a symptom-relevant gene set, genes associated with representative clinical symptoms of AS (e.g., inflammatory back pain, morning stiffness, peripheral arthritis) were screened from GeneCards (https://genecards.org/, accessed on 5 September 2025) using a relevance score threshold of ≥20. Firstly, symptoms regarding AS were selected based on the WHO ICD-11 framework and ASAS classification criteria. Representative clinical symptoms were defined a priori based on established clinical descriptions of ankylosing spondylitis and axial spondyloarthritis, with emphasis on manifestations directly reflecting patient-reported disease burden. Among potential manifestations, extra-articular features such as uveitis were deliberately excluded, as the present analysis focused on symptom domains directly related to musculoskeletal involvement. Each symptom term was treated as an independent query in GeneCards, and symptom–gene associations were retrieved separately rather than inferred from a single disease-level query. Symptom-associated gene lists were subsequently merged using a union-based strategy to preserve symptom-specific molecular diversity. This approach was intentionally adopted to avoid collapsing heterogeneous symptom signals into a single disease-centric profile prior to network-based refinement. The relevance score threshold of ≥20 was applied to prioritize gene–symptom associations supported by multiple evidence sources integrated within GeneCards and to reduce the inclusion of weak or low-confidence links. For comparison, AS-associated genes were retrieved from GeneCards using the keyword ‘ankylosing spondylitis’. Genes with a relevance score ≥ 20 were selected to ensure robust association. Each symptom term was queried independently in GeneCards, and symptom-associated gene lists were merged using a union-based strategy prior to network refinement. This procedure was adopted to preserve symptom-specific molecular signals and to minimize bias toward generic inflammatory gene enrichment.

### 2.2. Centrality Analysis

Centrality metrics were calculated for each node (gene) within the symptom-associated gene network using Netminer 4 software (Cyram Inc., Gyeonggi-do, Republic of Korea). Five network centrality indices were employed: Degree, Betweenness, Closeness, Eigenvector (Edge), and Eigenvector (Linked). Degree, betweenness, and closeness centrality were used to capture local connectivity, mediating roles, and global accessibility of genes within the network, respectively. Eigenvector centrality (linked and edge-based) was applied to identify genes with influence amplified by connections to other highly central nodes. Genes ranking in the top 5% for each index were selected, and those overlapping across multiple indices were identified as core symptom-associated genes. The use of a top 5% threshold across multiple centrality measures was intended to reduce bias toward single-metric universal hubs and to identify genes that consistently occupied influential positions within the symptom-associated network. After excluding low-confidence or disconnected nodes from the network, the remaining genes were defined as the core symptom-associated gene set. The disease-associated gene set was mapped to STRING (https://string-db.org/, version 12.0) with a confidence score threshold of 0.7 (Homo sapiens) to construct a protein–protein interaction network. Degree, betweenness, and closeness centrality were calculated as described for the symptom network.

### 2.3. Gene Expression Pattern Analysis

To validate the biological relevance of the identified core genes, gene expression patterns were analyzed using patient data. The NCBI Gene Expression Omnibus (GEO) dataset GDS5231 (Ankylosing spondylitis: blood) [30], which includes microarray data from AS patients and healthy controls, was selected. Fold changes in gene expression between AS and control samples were calculated for each probe to assess differential expression.

### 2.4. Network Proximity Analysis

A protein–protein interaction (PPI) network was constructed to evaluate the network-based proximity between the therapeutic target gene *TNF* and the 145 core genes. PPI data were obtained from the STRING database (https://string-db.org/, version 12.0) with a confidence score threshold of 0.7 for Homo sapiens. The STRING confidence threshold was applied to balance network completeness with reliability, ensuring exclusion of low-confidence interactions while retaining biologically supported connections relevant to symptom-level analysis. The PPI network was visualized using Cytoscape software (version 3.10.3) and STRING App (v.2.2.0) (data source: “STRING: protein query”). The shortest path lengths between *TNF* and each core gene were computed using the NetworkX Python package (version 3.13). The observed average shortest path was defined as the network proximity score.

### 2.5. Functional Enrichment Analysis

To capture genes most functionally relevant to drug–symptom interactions, we merged 13 genes directly connected to TNF with 14 highly connected genes from the top 10% of the 145 core genes based on network centrality. After removing redundancies, a total of 24 unique genes were selected. This gene set was then subjected to pathway enrichment using the Reactome Pathway Database (Version 94) (https://reactome.org/, accessed on 8 September 2025), allowing us to identify biologically significant immune and inflammatory signaling pathways associated with AS. To evaluate concordance between frameworks, the overlap between the symptom-core and disease-core gene sets was identified. The intersecting genes were subjected to STRING analysis to confirm connectivity and were also analyzed for functional enrichment using the ShinyGO v0.82 (https://bioinformatics.sdstate.edu/go/, accessed on 8 September 2025).

### 2.6. Statistics

All quantitative analyses and statistical evaluations were conducted as follows. For differential gene expression, a two-tailed *t*-test was applied to compare expression levels between AS patients and healthy controls using GDS5231 data, with fold change used to represent expression differences. A threshold of *p* < 0.05 was considered statistically significant. For network proximity analysis, statistical testing was performed by comparing the observed mean shortest path distance between TNF and the core gene set to a null distribution generated from 10,000 randomly sampled gene sets. The observed proximity was standardized using z-scores calculated from the mean and standard deviation of the null distribution. Corresponding *p*-values were derived from the cumulative distribution function of the standard normal distribution, with statistical significance set at *p* < 0.05. Reactome enrichment results were evaluated using false discovery rate (FDR) values, and pathways with FDR < 0.05 were considered significantly enriched.

## 3. Results

### 3.1. Limitations of Disease-Centric Network Analysis

**Figure 1:**
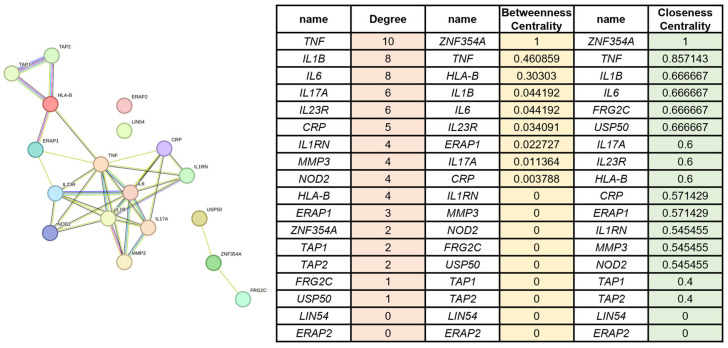
**Disease-centric gene network and centrality metrics.** Protein–protein interaction (PPI) network of AS-associated genes retrieved from GeneCards (relevance ≥ 20). Nodes represent genes, and edges represent high-confidence interactions (STRING score ≥ 0.7). Centrality analysis identified key nodes including *TNF*, *IL1B*, *IL6*, *IL17A*, and *IL23R*.

**Figure 2:**
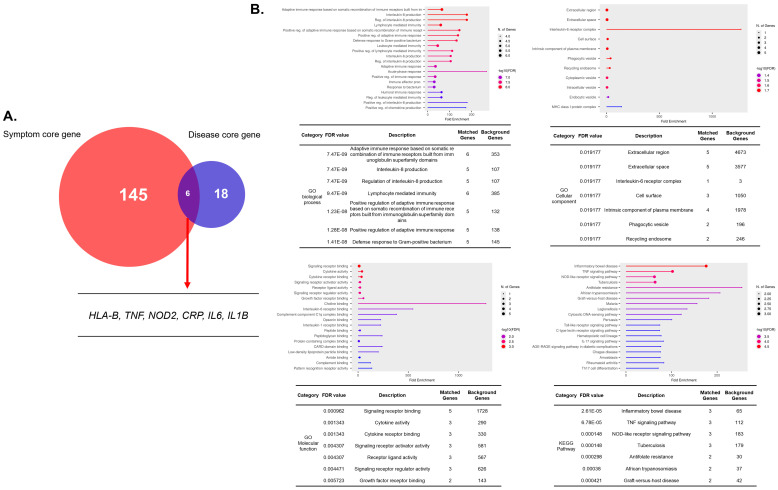
**Overlap between symptom-core and disease-core gene sets and functional enrichment of disease-core genes.** (**A**) Venn diagram showing limited overlap (*n* = 6) between the 145 symptom-core genes and the 18 disease-core genes. Shared genes included *HLA-B*, *TNF*, *NOD2*, *CRP*, *IL6*, and *IL1B*. (**B**) Gene Ontology (GO) and KEGG enrichment of the overlapped gene set. Functional categories were largely confined to broad immune-related processes, including adaptive immune response, cytokine activity, and inflammatory signaling.

To provide a disease-level reference, we also constructed a core gene network from AS-associated genes curated in GeneCards (relevance ≥ 20). This analysis yielded an 18-gene core dominated by canonical hubs such as *TNF*, *IL1B*, *IL6*, *IL17A*, *IL23R*, *CRP*, *IL1RN, MMP3*, *NOD2*, *HLA-B*, *ERAP1*, *ZNF354A*, *TAP1*, *TAP2*, *FRG2C*, *USP50*, *LIN54*, and *ERAP2* (Figure 1). Centrality assessment confirmed these nodes as the most connected and influential within the disease network. However, overlap with the 145 symptom-core genes was limited to only six shared nodes (*HLA-B*, *TNF*, *NOD2*, *CRP*, *IL6*, and *IL1B*) (Figure 2A). Functional enrichment of the disease-core genes resulted in a restricted set of broad immune-related pathways (Figure 2B).

### 3.2. Identification of Core Symptom Genes Through Centrality and PPI Analysis

**Figure 3:**
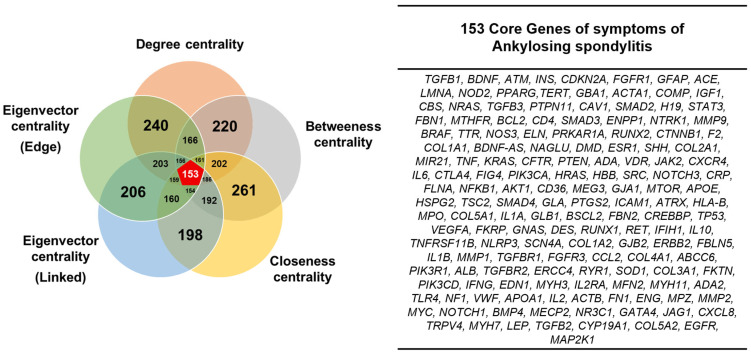
**Identification of core symptom-associated genes by multi-centrality analysis.** Venn diagram showing overlap among the top 5% of genes ranked by five centrality indices (degree, betweenness, closeness, eigenvector edge, and eigenvector linked). A total of 153 genes were consistently ranked highly across all measures, which were validated through PPI analysis to yield a final set of 145 core symptom-associated genes.

Network centrality analysis was performed using five different metrics, which included degree, betweenness, closeness, eigenvector linked, and eigenvector edge (Table 1). From the top 5% rankings of each metric, a total of 507 unique genes were identified. Among these, 372 genes were found to be ranked within the top 5% in at least two centrality measures. Applying a stricter criterion, 153 genes that consistently ranked within the top 5% across all 5 centrality indices were selected (Figure 3). After validating the network using a PPI analysis confidence score threshold of 0.7 and removing nodes with low confidence or no connectivity, 145 genes were finalized as the core symptom-associated gene set.

### 3.3. Expression Validation of Core Symptom-Associated Genes

**Figure 4:**
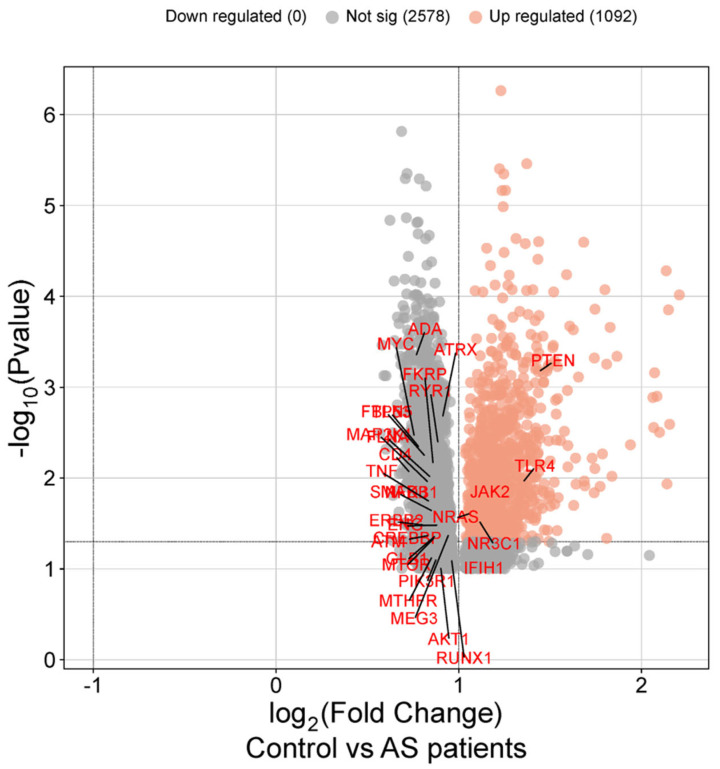
**Validation of core symptom-associated genes using patient transcriptomic data.** Volcano plot of differential gene expression in AS patients versus healthy controls (GDS5231 dataset). Red dots indicate upregulated genes, while gray dots represent non-significant changes. Five genes, *PTEN*, *TLR4*, *JAK2*, *NRAS*, and *NR3C1*, were significantly upregulated in AS patients, supporting their biological relevance.

Differential expression analysis was performed with adjustment for multiple testing using the Benjamini–Hochberg false discovery rate (FDR) procedure. Both nominal *p*-values and FDR-adjusted *p*-values were calculated, and effect size estimates were used to facilitate interpretation of biological relevance. Among these, five genes (*PTEN*, *TLR4*, *JAK2*, *NRAS*, and *NR3C1*) showed nominally increased expression in AS patients compared to healthy controls (Figure 4). *PTEN* exhibited a fold change of 1.436 (nominal *p* = 0.000678), followed by *TLR4* (fold change = 1.349, nominal *p* = 0.011243), *JAK2* (fold change = 1.285, nominal *p* = 0.012802), *NRAS* (fold change = 1.067, nominal *p* = 0.024315), and *NR3C1* (fold change = 1.109, nominal *p* = 0.028972).

### 3.4. Network Proximity Analysis of TNF Within the Symptom Gene Landscape

TNF was found to be directly connected to 13 genes, namely *TNF*, *HLA-B*, *IL10*, *PTPN11*, *AKT1*, *NTRK1*, *CTNNB1*, *BDNF*, *CTLA4*, *MYC*, *CD36*, *ACTB*, and *NOTCH1*, among the 145 core symptom-associated genes, representing 9.0 percent of the network. The average shortest path length between *TNF* and all core genes was calculated as 1.077. To assess statistical significance, this value was compared against a null distribution generated from 10,000 randomly sampled gene sets. The observed proximity produced a z-score of −0.009 and a *p*-value of 0.496, indicating that *TNF* does not show statistically significant global proximity to the core symptom-associated gene set. These results indicate that TNF exhibits prominent local connectivity within the symptom-associated network, despite the absence of statistically significant global proximity across the full gene set.

### 3.5. Pathway Enrichment of TNF-Linked and Central Symptom-Associated Genes

**Figure 5:**
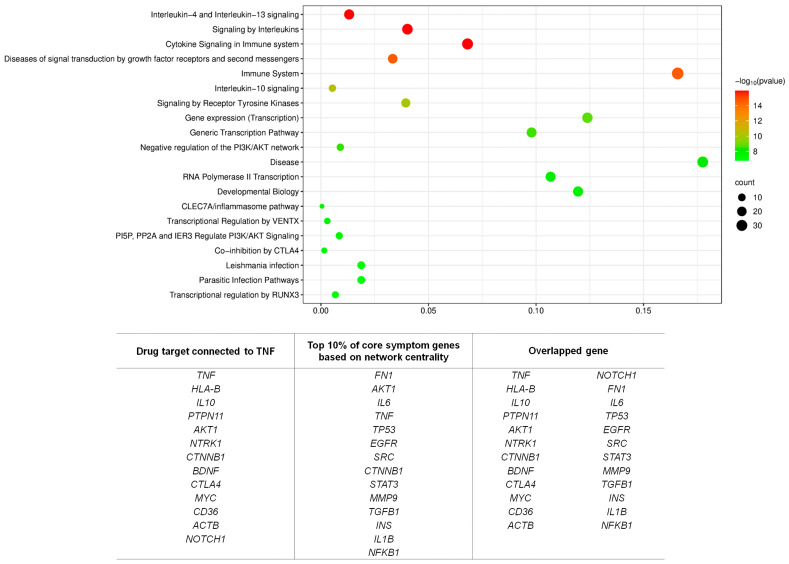
**Pathway enrichment of TNF-linked and central symptom-associated genes.** Bubble plot showing Reactome pathway enrichment of a refined 24-gene set, combining 13 TNF-linked neighbors and 14 highly central nodes from the symptom network. Significantly enriched pathways included interleukin signaling, cytokine signaling in the immune system, and interleukin-10 signaling.

13 genes directly linked to *TNF* and 14 genes ranked within the top 10% in network centrality among the 145 core symptom-associated genes were first selected. The 14 genes with high network centrality were *FN1*, *AKT1*, *IL6*, *TP53*, *EGFR*, *SRC*, *CTNNB1*, *STAT3*, *MMP9*, *TGFB1*, *INS*, *IL1B*, *NFKB1*, and *MYC*. After removing overlapping entries such as *AKT1*, *CTNNB1*, and *MYC*, which appeared in both groups, a final set of 24 unique genes was constructed. This 24-gene set was then subjected to Reactome pathway enrichment analysis, which identified several statistically significant pathways primarily related to immune function and inflammatory signaling. Notably, the most significantly enriched pathways included Interleukin-4 and Interleukin-13 signaling, Cytokine signaling in the immune system, and Interleukin-10 signaling, all showing high −log_10_(*p*-value) scores above 14 (Figure 5).

## 4. Discussion

The present study demonstrates the utility of a novel symptom-centered network pharmacology framework to explore the molecular basis of AS. By diverging from conventional disease-centric models, which often fail to explain clinical variability, our symptom-level approach successfully captured the molecular heterogeneity of AS with greater granularity. This strategy provides a more precise framework for aligning molecular dysregulation with specific patient-level manifestations, offering a promising path toward precision therapeutics.

The first major finding was the identification of 145 core symptom-associated genes through a combination of centrality analysis and PPI network refinement. Symptom-associated genes were not selected based on symptom frequency or keyword overlap but were prioritized through multi-metric network centrality, minimizing bias from database-driven parameter choices. The biological relevance of this gene set was further supported by validation against the GDS5231 dataset, which revealed significant upregulation of five genes, including *PTEN*, *TLR4*, *JAK2*, *NRAS*, and *NR3C1* in AS patients compared with healthy controls. These genes represent critical mechanistic anchors that bridge molecular dysregulation with clinical manifestations. *TLR4*, a central mediator of innate immunity, is activated by microbial antigens and reinforces the hypothesis that gut dysbiosis contributes to AS pathogenesis [31]. *JAK2* is a key component of the IL-23/Th17 axis, a pathway central to AS inflammation and directly targeted by recently approved JAK inhibitors [32]. *PTEN* and *NRAS* link inflammatory signaling to processes of cell survival and tissue remodeling, suggesting that structural changes in AS may involve pathways beyond cytokine-driven inflammation alone [33]. Finally, *NR3C1*, encoding the glucocorticoid receptor, provides a plausible molecular explanation for the variability in corticosteroid responsiveness observed in clinical practice [34]. Accordingly, the transcriptomic validation should be interpreted as supportive evidence linking network-derived candidates to patient data, rather than as definitive confirmation of disease-specific expression changes.

Beyond gene identification and expression validation, we investigated the network proximity of FDA-approved drug targets to the 145 symptom-associated genes. This analysis was motivated by the recognition that while TNF inhibitors remain the cornerstone of AS therapy, up to 30% of patients exhibit primary or secondary non-responsiveness [35]. Our results showed that *TNF* occupies a hub-like position within the symptom gene network and is directly connected to 13 of the identified core genes. These local connections emphasize the capacity of *TNF* inhibition to modulate critical immune-regulatory hubs that shape symptom expression. However, the lack of statistically significant global proximity between *TNF* and the entire 145-gene set suggests that the therapeutic effects of TNF inhibitors arise from selective rather than widespread network modulation. In contrast, the targets of more recently approved biologics, including IL-17A and JAK inhibitors, were not represented within the symptom-associated network. This finding provides a plausible molecular explanation for the clinical observation that while many patients benefit substantially from *TNF* blockade, others experience only partial or no improvement [15]. The targets of more recently approved biologics, including IL-17A and JAK inhibitors, were not represented within the symptom-associated network. Although IL-17 and JAK pathways are well-established drivers of AS pathogenesis at the disease level, their absence from the symptom-level gene set may account for the variable treatment responses observed in clinical practice. To address this complexity, we refined our analysis by constructing a 24-gene set that integrated two complementary perspectives: 13 genes directly connected to *TNF* within the symptom-associated network and 14 genes from the top 10% of centrality rankings among the 145 core genes. This combined set captured both the immediate pharmacological neighborhood of *TNF* and the broader symptom-related hubs. Enrichment analysis of the 24-gene set revealed significant associations with immune and inflammatory signaling pathways, including Interleukin-4 and Interleukin-13 signaling, Cytokine signaling in the immune system, and Interleukin-10 signaling. These pathways are highly relevant to AS, as they regulate processes such as T-helper cell differentiation, cytokine-mediated communication, and anti-inflammatory feedback. The convergence of *TNF*-linked and symptom-central genes on these pathways suggests that symptom expression in AS is coordinated by broader immune-modulatory circuits rather than by isolated molecular targets. Within the context of an immune-mediated disease such as ankylosing spondylitis, enrichment of immune and cytokine signaling pathways is biologically expected. Importantly, from a symptom-centered perspective, the clinical relevance of the 24-gene set lies not in pathway novelty but in its ability to link immune regulatory circuits to specific patient-reported symptom manifestations. By integrating TNF-linked genes with highly central symptom-associated nodes, this focused module captures immune pathways that are most directly aligned with patient-reported disease burden, rather than representing a nonspecific inflammatory signature.

This interpretation offers several important insights. First, it provides a molecular rationale for the broad clinical efficacy of TNF inhibitors, despite the absence of statistically significant global proximity: their direct influence on key hubs within immune-inflammatory circuits may be sufficient to disrupt symptom-driving processes. Second, it helps explain the heterogeneity of responses to IL-17A and JAK inhibitors, as these targets may not consistently overlap with the symptom-centric networks that drive clinical manifestations. Third, the integration of TNF-linked and highly central symptom-associated genes highlights potential opportunities for adjunctive or alternative targets that could complement the limitations of current biologics. Previous investigations of AS have typically focused on disease-centric gene sets identified from genome-wide association studies or transcriptomic profiling of patient cohorts [36]. These studies consistently emphasized the roles of HLA-B27, IL-23/Th17 signaling, and bone remodeling pathways, which remain critical to understanding AS pathology [37]. However, by collapsing the disease into a single set of genes, such approaches may obscure the molecular heterogeneity underlying distinct symptom domains. Symptom-centered analysis, by contrast, dissected the molecular correlates of specific clinical manifestations such as inflammatory back pain, morning stiffness, and peripheral arthritis, thereby offering a perspective on how pathogenesis translates into patient experience. It is important in the context of personalized medicine, where the alignment between therapeutic targets and patient-specific symptomatology may determine treatment success or failure.

The multi-step analytical framework adopted in this study, which combined relevance thresholds, multi-metric centrality analysis, expression validation, and drug–symptom proximity assessment, allowed us to minimize spurious associations and enhance the biological reliability of our findings. Importantly, clinical symptoms were explicitly defined and independently operationalized prior to gene collection, allowing symptom-associated molecular signals to be distinguished from generic inflammatory or pain-related biology. A limitation of this study is the reliance on a single integrative database for gene collection, which may bias the analysis toward well-studied targets. However, this potential limitation was partially mitigated by the subsequent multi-layered network refinement strategy. In particular, refining the network into a 24-gene set by integrating *TNF*-linked and highly central nodes provided a clearer interpretive landscape, ensuring that enrichment results were both mechanistically grounded and clinically meaningful. Even results that did not reach statistical significance in proximity testing contributed valuable insights when considered in the context of clinical heterogeneity, highlighting the selective rather than global influence of existing therapies. At the same time, certain aspects should be interpreted with caution. The reliance on whole-blood transcriptomic data means that expression changes in primary sites of pathology, such as the sacroiliac joints or entheses, may not have been fully captured. Protein–protein interaction data also reflect only currently annotated relationships, potentially missing tissue-specific or dynamic interactions. In addition, the proximity analysis was limited to *TNF*, as it was the only FDA-approved target overlapping with the symptom network, and therefore does not encompass the full spectrum of therapeutic agents currently used in AS. To further advance these findings, incorporating tissue-specific omics data from inflamed spinal and entheseal sites could provide a more accurate representation of AS pathology. Expanding the analytical framework to include proteomic and epigenetic layers may also clarify how symptom-associated networks are regulated across multiple biological dimensions. In addition, module-based analyses of the identified network could reveal whether distinct clusters of genes correspond to particular symptom domains, thereby informing strategies for more targeted therapeutic interventions. Moreover, extending proximity analyses to include IL-17A and JAK inhibitor targets may also help explain the heterogeneity of treatment responses observed in clinical settings.

While traditional disease-centric frameworks have contributed foundational insights into AS pathogenesis, our findings suggest that they are insufficient to fully capture the molecular complexity of the disease. Enrichment analysis of the disease-core gene set yielded only broad immune-related categories with limited specificity, and its minimal overlap with the symptom-core network highlights the limitations of collapsing clinical heterogeneity into a single gene set. In contrast, the symptom-centric approach preserved pathway diversity and identified immune-regulatory circuits more directly associated with symptom expression. This framework may therefore serve as a more precise and clinically relevant strategy for delineating AS heterogeneity and developing symptom-specific therapeutic interventions. In conclusion, this study demonstrates that while our findings provide novel insights into the molecular architecture of AS symptoms, they also reveal areas where future research is needed to refine and extend the approach. By continuing to align molecular networks with clinical manifestations, symptom-centered network pharmacology may contribute to therapeutic strategies that are not only biologically precise but also better tailored to the lived heterogeneity of AS patients.

## Figures and Tables

**Table 1:** Network centrality analysis.

Top 5% Gene	Degree Score	Top 5% Gene	Betweenness Score	Top 5% Gene	Closeness Score	Top 5% Gene	Eigenvector (Linked) Score	Top 5% Gene	Eigenvector (Edge) Score
*TNF*	0.875	*TNF*	0.063414	*TGFB1*	0.996747	*TGFB1*	0.028799	*TP53*	0.093697
*IL6*	0.833333	*IL17A*	0.056259	*LMNA*	0.996406	*LMNA*	0.028618	*TGFB1*	0.068743
*TGFB1*	0.833333	*NOD2*	0.05239	*FBN1*	0.996065	*CBS*	0.028142	*PTEN*	0.066873
*LMNA*	0.791667	*SGSH*	0.042887	*BRAF*	0.995895	*FBN1*	0.028111	*LMNA*	0.063758
*TP53*	0.75	*IL17RA*	0.039644	*CBS*	0.995895	*BRAF*	0.028103	*IL6*	0.063713
*FBN1*	0.75	*IL36RN*	0.0394	*TNF*	0.995724	*COL1A1*	0.028085	*TNF*	0.062645
*IL10*	0.708333	*FBN1*	0.026806	*COL1A1*	0.995554	*TNF*	0.028057	*PIK3CA*	0.062082
*IL1B*	0.708333	*LTA*	0.02657	*NF1*	0.994534	*IL6*	0.028036	*BRAF*	0.060777
*CRP*	0.708333	*CARD14*	0.026201	*ELN*	0.994364	*CRP*	0.027805	*FGFR3*	0.060252
*HLA-DRB1*	0.708333	*MIR146A*	0.023174	*PTPN11*	0.994194	*APOE*	0.027788	*AKT1*	0.060211
*BRAF*	0.708333	*IL23R*	0.022281	*MMP2*	0.993515	*HLA-B*	0.02774	*EGFR*	0.059777
*CBS*	0.708333	*TGFB1*	0.02178	*IL6*	0.993007	*TP53*	0.027735	*FBN1*	0.058401
*IFNG*	0.666667	*HLA-C*	0.021439	*IL17A*	0.993007	*IL10*	0.027694	*ATM*	0.057729
*HLA-B*	0.666667	*LMNA*	0.021071	*IL10*	0.992499	*IL1B*	0.027556	*RYR1*	0.05748
*H19*	0.666667	*IL6*	0.020554	*PTEN*	0.992499	*ALB*	0.027514	*INS*	0.055908
*NF1*	0.666667	*BRAF*	0.020282	*PIK3CA*	0.992499	*IFNG*	0.027505	*NF1*	0.055527
*COL1A1*	0.666667	*CBS*	0.020159	*AKT1*	0.992499	*NF1*	0.027405	*KRAS*	0.05526
*CXCL8*	0.625	*NF1*	0.019314	*SMAD4*	0.992499	*MMP2*	0.027377	*COL2A1*	0.055182
*EGFR*	0.625	*PTPN11*	0.018886	*NOTCH3*	0.992499	*CXCL8*	0.027376	*COL1A1*	0.054745
*TLR4*	0.625	*IL10*	0.018678	*BMP4*	0.992499	*TLR4*	0.027344	*APOE*	0.053907
*STAT3*	0.625	*COL1A1*	0.018295	*CRP*	0.99233	*ACE*	0.02734	*CTNNB1*	0.053261
*IL1A*	0.625	*TP53*	0.018288	*ALB*	0.991992	*IGF1*	0.02734	*HRAS*	0.052865
*IL17A*	0.625	*ELN*	0.017428	*ACE*	0.991992	*STAT3*	0.027336	*ACE*	0.052721
*ALB*	0.625	*CRP*	0.017164	*IGF1*	0.991992	*MMP9*	0.027336	*STAT3*	0.052613
*TERT*	0.625	*IL1B*	0.016873	*TP53*	0.991823	*F2*	0.027322	*TERT*	0.05245
*MMP9*	0.625	*ACE*	0.016648	*F2*	0.991823	*INS*	0.027188	*IGF1*	0.052366
*APOE*	0.625	*IGF1*	0.016648	*APOE*	0.991485	*TERT*	0.027144	*IL10*	0.052192
*ACE*	0.625	*TGFBR2*	0.016532	*IL1B*	0.991316	*PTPN11*	0.027051	*SMAD4*	0.051937
*KRAS*	0.625	*ALB*	0.016401	*IFNG*	0.991147	*CD4*	0.027035	*NTRK1*	0.051661
*IGF1*	0.625	*F2*	0.016326	*CXCL8*	0.990979	*ELN*	0.026904	*BRCA2*	0.051291
*CD4*	0.625	*BMP4*	0.016245	*TLR4*	0.990979	*DMD*	0.026834	*SQSTM1*	0.051186
*F2*	0.625	*MMP2*	0.016211	*STAT3*	0.990979	*PTEN*	0.026816	*SOD1*	0.050498
*FGFR3*	0.625	*PTEN*	0.016125	*MIR21*	0.990979	*PIK3CA*	0.026816	*FLNA*	0.049436
*PTPN11*	0.625	*PIK3CA*	0.016125	*MMP9*	0.990979	*AKT1*	0.026816	*NOTCH1*	0.049432
*NTRK1*	0.583333	*AKT1*	0.016125	*GLB1*	0.990979	*SMAD4*	0.026816	*CBS*	0.049166
*NOD2*	0.583333	*SMAD4*	0.016125	*TERT*	0.99081	*IL1A*	0.026793	*VCP*	0.047156
*BDNF-AS*	0.583333	*NOTCH3*	0.015898	*HLA-B*	0.990641	*IL17A*	0.026652	*FGFR2*	0.046912
*IL1RN*	0.583333	*MIR21*	0.015825	*IL1A*	0.990473	*GNAS*	0.026591	*PTPN11*	0.046295
*PTEN*	0.583333	*FGFR3*	0.015812	*INS*	0.990473	*SCN4A*	0.026585	*IL1B*	0.046293
*MIR21*	0.583333	*IFNG*	0.01579	*ICAM1*	0.990473	*FGFR3*	0.026564	*BRCA1*	0.046259
*PIK3CA*	0.583333	*APOE*	0.015527	*IL2RA*	0.990136	*RYR1*	0.026534	*APP*	0.046022
*CTNNB1*	0.583333	*STAT3*	0.015489	*IL2*	0.990136	*IL2RA*	0.026501	*MAPT*	0.045921
*AKT1*	0.583333	*MMP9*	0.015489	*DMD*	0.990136	*IL2*	0.026501	*MTHFR*	0.045736
*INS*	0.583333	*TLR4*	0.015463	*NOS3*	0.989968	*BMP4*	0.026437	*DMD*	0.045493
*TTR*	0.583333	*TERT*	0.015418	*EDN1*	0.989968	*LEP*	0.026425	*CDKN2A*	0.045463
*TGFBR2*	0.583333	*IL1A*	0.015385	*VWF*	0.989968	*EGFR*	0.026395	*BMP4*	0.045174
*COL2A1*	0.583333	*ICAM1*	0.015376	*BDNF-AS*	0.989631	*GFAP*	0.026371	*IFNG*	0.045096
*CERNA3*	0.583333	*CXCL8*	0.015368	*TGFBR2*	0.989631	*COMP*	0.026289	*FGFR1*	0.044917
*SMAD4*	0.583333	*CAV1*	0.015334	*CAV1*	0.989463	*H19*	0.026247	*ELN*	0.043889
*MMP2*	0.583333	*EGFR*	0.015207	*EGFR*	0.989295	*NTRK1*	0.026225	*CEP290*	0.043826
*HRAS*	0.583333	*NOS3*	0.015091	*GNAS*	0.989295	*CTNNB1*	0.026215	*CFTR*	0.043625
*ELN*	0.583333	*HLA-B*	0.015047	*SCN4A*	0.989127	*COL2A1*	0.026177	*COL1A2*	0.042618
*BMP4*	0.583333	*KRAS*	0.015042	*MIR223*	0.989127	*BDNF*	0.026176	*H19*	0.042452
*NGF*	0.541667	*PPARG*	0.014997	*SMAD3*	0.989127	*NOD2*	0.026163	*TRPV4*	0.042259
*TNFRSF1A*	0.541667	*EDN1*	0.01488	*ABCC6*	0.989127	*NRAS*	0.026125	*CHEK2*	0.042243
*CCL2*	0.541667	*VWF*	0.01488	*CTNNB1*	0.988959	*MTHFR*	0.026096	*NRAS*	0.041919
*BDNF*	0.541667	*GNAS*	0.014846	*PPARG*	0.988959	*CSF3*	0.026083	*TGFBR2*	0.041912
*IL2RA*	0.541667	*INS*	0.014793	*RYR1*	0.988959	*TTR*	0.026074	*ESR1*	0.041776
*IL2*	0.541667	*COL2A1*	0.014736	*COMP*	0.988959	*BDNF-AS*	0.026058	*BDNF-AS*	0.041421
*SCN4A*	0.541667	*BDNF-AS*	0.014651	*NOD2*	0.988791	*KRAS*	0.026042	*CDH1*	0.041069
*PPARG*	0.541667	*IL2RA*	0.014645	*FGFR3*	0.988791	*CERNA3*	0.02602	*PALB2*	0.040956
*ICAM1*	0.541667	*IL2*	0.014645	*CD36*	0.988791	*CTLA4*	0.026012	*SCN4A*	0.040686
*TRPV4*	0.541667	*CTNNB1*	0.014583	*MPO*	0.988623	*FLNA*	0.025935	*GNAS*	0.040438
*MPZ*	0.541667	*GLB1*	0.014549	*TGFBR1*	0.988623	*HSPG2*	0.025935	*NOD2*	0.040004
*ATM*	0.541667	*SMAD3*	0.014456	*FLNA*	0.988623	*MPO*	0.02593	*BDNF*	0.039969
*SOD1*	0.541667	*DMD*	0.014393	*HSPG2*	0.988623	*VEGFA*	0.025894	*APC*	0.039844
*MMP1*	0.541667	*TGFBR1*	0.014379	*MFN2*	0.988455	*TNFRSF11B*	0.025886	*RET*	0.039579
*LEP*	0.541667	*MMP1*	0.014144	*ACTC1*	0.988455	*MMP1*	0.025865	*HLA-B*	0.039426
*CDKN2A*	0.541667	*SCN4A*	0.014117	*NOS2*	0.988287	*TGFBR2*	0.025849	*TTR*	0.039226
*CSF3*	0.541667	*MIR223*	0.014087	*ADA*	0.988287	*EDN1*	0.025847	*MYH7*	0.039081
*TNFRSF11B*	0.541667	*NOS2*	0.014045	*MMP1*	0.988287	*VWF*	0.025847	*CTLA4*	0.038822
*MTHFR*	0.541667	*SRC*	0.013876	*COL1A2*	0.988287	*MYC*	0.02582	*PPARG*	0.038747
*VEGFA*	0.541667	*ACTC1*	0.013866	*ELANE*	0.988119	*TRPV4*	0.025808	*MTOR*	0.038728
*GNAS*	0.541667	*ELANE*	0.013851	*SRC*	0.988119	*MPZ*	0.025808	*ALB*	0.038546
*DMD*	0.541667	*MPO*	0.01383	*TGFB2*	0.988119	*MIR21*	0.025801	*TTN*	0.038367
*NOS3*	0.541667	*ADA*	0.013828	*FBN2*	0.988119	*IL1RN*	0.0258	*CRP*	0.038336
*NRAS*	0.541667	*NOTCH1*	0.013811	*TGFB3*	0.987952	*CXCR4*	0.025796	*CREBBP*	0.038064
*GBA1*	0.541667	*TGFB2*	0.013759	*NOTCH1*	0.987952	*NOTCH3*	0.025795	*GBA1*	0.03798
*FGFR1*	0.541667	*DSP*	0.01359	*HMOX1*	0.987784	*MTOR*	0.025729	*TLR4*	0.037887
*PTGS2*	0.5	*CD36*	0.013569	*COL2A1*	0.987784	*ATRX*	0.025729	*SHH*	0.037821
*COMT*	0.5	*ABCC6*	0.01354	*ATRX*	0.987784	*CDKN2A*	0.025726	*MECP2*	0.037728
*NFKB1*	0.5	*COL1A2*	0.01351	*PRKAR1A*	0.987617	*TNFRSF1A*	0.025724	*POLG*	0.037696
*IL4*	0.5	*GLRA1*	0.013501	*CCN2*	0.987617	*GBA1*	0.025723	*MAP2K1*	0.037672
*CTLA4*	0.5	*CD4*	0.013412	*ACTA1*	0.987449	*CAV1*	0.025718	*GFAP*	0.037535
*ELANE*	0.5	*CCN2*	0.013377	*DSP*	0.987449	*SMAD3*	0.02563	*MIR21*	0.037499
*CD40LG*	0.5	*NTRK1*	0.013368	*FKTN*	0.987449	*PRKAR1A*	0.025606	*DYSF*	0.037489
*HLA-DQB1*	0.5	*HMOX1*	0.013367	*PTGS2*	0.987282	*ESR1*	0.025603	*GLB1*	0.037471
*RYR1*	0.5	*PTGS2*	0.013283	*CD4*	0.987114	*ADA*	0.025572	*SOX9*	0.037466
*GJB1*	0.5	*FBN2*	0.013188	*SMAD2*	0.986947	*HRAS*	0.025553	*ERBB2*	0.037256
*JAK2*	0.5	*COMP*	0.013053	*IL5*	0.98678	*CD36*	0.025547	*TSC2*	0.037195
*ADA*	0.5	*RYR1*	0.01305	*LEP*	0.98678	*GLA*	0.025495	*HSPG2*	0.037161
*MPO*	0.5	*GBA1*	0.013018	*FGF2*	0.98678	*GLB1*	0.025495	*BRIP1*	0.03709
*CFTR*	0.5	*LEP*	0.013014	*GJB2*	0.986612	*DES*	0.025472	*MYC*	0.037002
*MIR223*	0.5	*ATRX*	0.013001	*TIMP1*	0.986612	*COL1A2*	0.025468	*IGF1R*	0.03679
*ESR1*	0.5	*H19*	0.012952	*MYH3*	0.986612	*CCL2*	0.025453	*SMAD3*	0.036744
*CXCR4*	0.5	*MTHFR*	0.012912	*TBX4*	0.986612	*SOD1*	0.025424	*SNCA*	0.03672
*FAS*	0.5	*MFN2*	0.012891	*GFAP*	0.986278	*MFN2*	0.025407	*ATRX*	0.036527
*MAPK1*	0.5	*IL5*	0.012851	*TPM3*	0.986278	*ACTB*	0.025404	*SRC*	0.036482
*MIR125A*	0.5	*FKTN*	0.012818	*RUNX2*	0.986278	*MECP2*	0.025403	*EZH2*	0.036427
*SMAD3*	0.5	*SMAD2*	0.012774	*NTRK1*	0.986278	*TGFB2*	0.025379	*MMP2*	0.036204
*SRC*	0.5	*BDNF*	0.012749	*SOD1*	0.986111	*MAP2K1*	0.025373	*MET*	0.036179
*GLA*	0.5	*FGF2*	0.012713	*MTHFR*	0.986111	*JAK2*	0.02537	*SCN9A*	0.036126
*TGFBR1*	0.5	*CXCL10*	0.012669	*DES*	0.986111	*MAPK1*	0.02537	*FLNC*	0.036034
*MAP2K1*	0.5	*FLNA*	0.012665	*CTLA4*	0.985944	*ELANE*	0.025347	*CXCL8*	0.035913
*MYC*	0.5	*HSPG2*	0.012665	*CXCL10*	0.985944	*SRC*	0.02534	*BMP2*	0.035792
*CDH1*	0.5	*MYH7*	0.012591	*APOA1*	0.985944	*GJA1*	0.025334	*SLC2A1*	0.035779
*TGFB2*	0.5	*PRKAR1A*	0.012564	*MBTPS2*	0.985944	*ICAM1*	0.02532	*FIG4*	0.035714
*BRCA1*	0.5	*APOA1*	0.012471	*PIK3C2A*	0.985944	*FBN2*	0.025306	*GJA1*	0.035535
*GFAP*	0.5	*SOD1*	0.01246	*RMRP*	0.985944	*RET*	0.02529	*MMP9*	0.035522
*APOA1*	0.5	*HRAS*	0.012441	*BDNF*	0.985777	*ATM*	0.025276	*ACTB*	0.035301
*EDN1*	0.5	*GJB2*	0.012406	*MYC*	0.985777	*PPARG*	0.025259	*MFN2*	0.035176
*MTOR*	0.5	*TIMP1*	0.012406	*BCL2*	0.985443	*IDH1*	0.02524	*TNFRSF11B*	0.035156
*VWF*	0.5	*TBX4*	0.012406	*ERCC4*	0.985277	*TGFB3*	0.025223	*PRKAR1A*	0.035136
*MFN2*	0.5	*IL1RN*	0.012321	*GLA*	0.98511	*BCL2*	0.025221	*COL5A1*	0.035118
*MYH7*	0.5	*TGFB3*	0.012296	*GJA1*	0.98511	*NOS3*	0.025208	*KIT*	0.035111
*CAV1*	0.5	*DES*	0.012266	*COL5A1*	0.984777	*NGF*	0.025206	*AR*	0.035081
*COMP*	0.5	*GLA*	0.012242	*CXCR4*	0.98461	*NAGLU*	0.025195	*GLA*	0.034793
*NOTCH3*	0.5	*JAG1*	0.01223	*CDKN2A*	0.98461	*CFTR*	0.025177	*NLRP3*	0.034642
*NOTCH1*	0.5	*RUNX2*	0.012116	*FBLN5*	0.98461	*FIG4*	0.025158	*ATP7A*	0.034636
*ATRX*	0.5	*ACTA1*	0.01208	*POLR3A*	0.98461	*NFKB1*	0.025153	*NOTCH3*	0.034536
*COL1A2*	0.5	*CDH1*	0.012049	*ESR1*	0.984444	*MIR125A*	0.025145	*TGFB3*	0.03436
*FGFR2*	0.5	*CTLA4*	0.012017	*ENPP1*	0.984444	*TSC2*	0.02513	*DES*	0.034339
*SCN9A*	0.458333	*COL5A1*	0.012013	*ABCC9*	0.984277	*COL5A1*	0.025109	*MAPK1*	0.034212
*MEFV*	0.458333	*GFAP*	0.01201	*PLG*	0.984277	*FKRP*	0.025104	*COL3A1*	0.034158
*NLRP3*	0.458333	*CDKN2A*	0.011983	*MBL2*	0.984277	*NLRP3*	0.025083	*SMARCA4*	0.034106
*ABCB1*	0.458333	*MYC*	0.01196	*INPP5E*	0.984277	*TGFBR1*	0.025079	*RUNX2*	0.034098
*TLR2*	0.458333	*GPHN*	0.011954	*VCAM1*	0.984111	*ERCC4*	0.02506	*IGF2*	0.033837
*HFE*	0.458333	*MYH3*	0.011879	*BSCL2*	0.984111	*CD40LG*	0.025011	*GCK*	0.033832
*IL1R1*	0.458333	*CXCR4*	0.011616	*SERPINC1*	0.984111	*MYH3*	0.025006	*PTCH1*	0.033637
*IL13*	0.458333	*BCL2*	0.0116	*NLRP3*	0.983945	*POLG*	0.024994	*RPGRIP1L*	0.033629
*IL18*	0.458333	*ESR1*	0.011558	*TRPV1*	0.983945	*VCP*	0.024994	*VEGFA*	0.033441
*MIR155*	0.458333	*TPM3*	0.011552	*IKBKG*	0.983945	*COMT*	0.02499	*PDGFRB*	0.033347
*RET*	0.458333	*CCL2*	0.011535	*CREBBP*	0.983945	*APOA1*	0.024944	*SH3TC2*	0.033297
*NOS2*	0.458333	*MBTPS2*	0.011519	*CALCA*	0.983778	*CCN2*	0.024912	*VWF*	0.033292
*IDH1*	0.458333	*PIK3C2A*	0.011519	*MIR34A*	0.983778	*NOTCH1*	0.024903	*ABCC8*	0.033291
*COL5A1*	0.458333	*RMRP*	0.011519	*COL3A1*	0.983778	*MYH7*	0.024887	*FKRP*	0.033222
*HMOX1*	0.458333	*TRPV1*	0.011496	*LOX*	0.983778	*IL18*	0.024881	*HNF4A*	0.033177
*CSF2*	0.458333	*DVL1*	0.0114	*MYH11*	0.983778	*DNMT1*	0.024881	*FBN2*	0.032917
*MMP3*	0.458333	*POLR3A*	0.011378	*TNXB*	0.983778	*JAG1*	0.02488	*ADAR*	0.032859
*BRCA2*	0.458333	*CERNA3*	0.011356	*PIK3R1*	0.983612	*CREBBP*	0.024843	*BCL2*	0.032827
*NAGLU*	0.458333	*ABCC9*	0.011333	*NAGLU*	0.983612	*IFIH1*	0.024841	*OFD1*	0.032701
*PRTN3*	0.458333	*GJA1*	0.011302	*PIK3CD*	0.983612	*FBLN5*	0.024814	*EP300*	0.0327
*CCND1*	0.458333	*PLG*	0.011301	*ERCC8*	0.983612	*BRCA1*	0.024811	*MLH1*	0.032656
*ERBB2*	0.458333	*MBL2*	0.011301	*STAT5A*	0.983612	*MAPT*	0.024804	*KCNJ11*	0.032656
*DNMT1*	0.458333	*INPP5E*	0.011301	*NR3C1*	0.983446	*GALNS*	0.024804	*F2*	0.032617
*MET*	0.458333	*MEG3*	0.011292	*IFIH1*	0.98328	*MIR223*	0.024795	*MSH2*	0.03252
*POLG*	0.458333	*ERCC4*	0.011291	*THBD*	0.98328	*PIK3R1*	0.024729	*CD4*	0.032489
*VCP*	0.458333	*NLRP3*	0.011258	*RUNX1*	0.98328	*PIK3CD*	0.024729	*IL2*	0.032465
*MECP2*	0.458333	*COL3A1*	0.011153	*SHH*	0.98328	*FGFR1*	0.024665	*PMM2*	0.032394
*CD36*	0.458333	*LOX*	0.011153	*IFNA1*	0.98328	*ACTA1*	0.024653	*GJB2*	0.032318
*TNFSF11*	0.458333	*MYH11*	0.011153	*ERBB2*	0.983114	*SMARCB1*	0.024641	*LEP*	0.032299
*TTN*	0.458333	*TNXB*	0.011153	*APOB*	0.983114	*SCN1A*	0.02464	*CXCR4*	0.032276
*FKRP*	0.458333	*ERBB2*	0.011117	*SST*	0.983114	*SMAD2*	0.024617	*MMP1*	0.032165
*DES*	0.458333	*F5*	0.011084	*PRKAG2*	0.983114	*ENPP1*	0.024589	*ENPP1*	0.032134
*TSC2*	0.458333	*ENPP1*	0.011034	*VCL*	0.983114	*POMC*	0.024527	*SDHB*	0.031936
*FIG4*	0.458333	*CSF3*	0.011014	*LDLR*	0.983114	*TTN*	0.024503	*TGFBR1*	0.031924
*FLNA*	0.458333	*FBLN5*	0.011006	*NEFL*	0.982948	*RUNX2*	0.024473	*PIK3R1*	0.031854
*ACTB*	0.458333	*NAGLU*	0.010967	*BAX*	0.982948	*AIFM1*	0.024445	*TH*	0.0318
*HSPG2*	0.458333	*LDLR*	0.010949	*SUMF1*	0.982948	*BRCA2*	0.024433	*POLR3A*	0.031722
*GJA1*	0.458333	*CREBBP*	0.010897	*F5*	0.982782	*MET*	0.024433	*GLI3*	0.03172
*BCL2*	0.458333	*PIK3R1*	0.010896	*TNFRSF1B*	0.982782	*SHH*	0.024423	*TSC1*	0.031661
*CD8A*	0.458333	*PIK3CD*	0.010896	*CASP3*	0.982782	*VDR*	0.024415	*IL2RA*	0.031571
*JAG1*	0.458333	*C9orf72*	0.010876	*SNCA*	0.982616	*HBB*	0.024415	*RAF1*	0.031544
*GLB1*	0.458333	*FGFR1*	0.010867	*SLC2A1*	0.982616	*TNFSF11*	0.02441	*ABCC6*	0.031492
*PRKAR1A*	0.458333	*VCAM1*	0.01086	*KCNQ2*	0.982616	*HMOX1*	0.024395	*TMEM67*	0.031465
*FBN2*	0.458333	*SHH*	0.010831	*DYSF*	0.982616	*SOX10*	0.024395	*ACTA1*	0.031448
*CCN2*	0.458333	*APOB*	0.010819	*LMNB1*	0.982616	*MEG3*	0.024373	*COMT*	0.031403
*SOX9*	0.458333	*PRKAG2*	0.010819	*CYP19A1*	0.982616	*PTGS2*	0.024365	*PRKN*	0.031393
*PDGFB*	0.458333	*VCL*	0.010819	*ITGB1*	0.982616	*SCN9A*	0.024332	*NGF*	0.031356
*SPP1*	0.458333	*IKBKG*	0.010817	*COL5A2*	0.982616	*MBTPS2*	0.024293	*MITF*	0.031353
*MEG3*	0.458333	*BSCL2*	0.010806	*ADA2*	0.98245	*PIK3C2A*	0.024293	*HNF1A*	0.031343
*SCN10A*	0.416667	*CCR5*	0.010783	*SOD2-OT1*	0.982284	*RMRP*	0.024293	*ERCC2*	0.031305
*PDGFRA*	0.416667	*SERPINC1*	0.010742	*ADRB2*	0.982284	*BSCL2*	0.024244	*LRP5*	0.031266
*TRPV1*	0.416667	*CASP3*	0.010741	*IGF1R*	0.982284	*SUMF1*	0.024243	*IFIH1*	0.031207
*IFIH1*	0.416667	*MIR125A*	0.010707	*GATA1*	0.982284	*SOX9*	0.024217	*CAV1*	0.031172
*STAT1*	0.416667	*ATM*	0.010701	*ITGB3*	0.982284	*TH*	0.024203	*INSR*	0.031145
*GCH1*	0.416667	*TNFRSF1B*	0.010689	*CD79A*	0.982284	*FGF2*	0.02418	*TUBB3*	0.031072
*IL5*	0.416667	*JAK2*	0.010672	*CLCN5*	0.982284	*FGFR2*	0.024176	*GAA*	0.030926
*CCR5*	0.416667	*MTOR*	0.010658	*SLC29A3*	0.982284	*RUNX1*	0.02413	*LDLR*	0.030898
*POMC*	0.416667	*CFTR*	0.010619	*SAMHD1*	0.982119	*IFNA1*	0.02413	*HBB*	0.03084
*VDR*	0.416667	*IFIH1*	0.010606	*AKT2*	0.982119	*SPP1*	0.024116	*JAG1*	0.030834
*PIK3R1*	0.416667	*THBD*	0.010591	*CCR5*	0.981953	*CTSK*	0.024091	*SMAD2*	0.030793
*VCAM1*	0.416667	*RUNX1*	0.010547	*DST*	0.981953	*FOS*	0.024088	*VDR*	0.030749
*HSPD1*	0.416667	*IFNA1*	0.010547	*SYNJ1*	0.981953	*NF2*	0.024066	*HLA-DRB1*	0.030591
*HBB*	0.416667	*CALCA*	0.010504	*HMGCR*	0.981953	*SLC17A5*	0.024041	*NOS3*	0.030536
*PIK3CD*	0.416667	*MIR34A*	0.010504	*STIM1*	0.981953	*ANO5*	0.024041	*FLNB*	0.030321
*FOS*	0.416667	*TRPV4*	0.0105	*C3*	0.981953	*GJB2*	0.024019	*SCN1A*	0.030128
*CXCL10*	0.416667	*MPZ*	0.0105	*GDNF*	0.981953	*TIMP1*	0.024019	*NEFL*	0.030085
*SCN1A*	0.416667	*CYP19A1*	0.010482	*KNG1*	0.981788	*TBX4*	0.024019	*NAGLU*	0.030047
*TNFRSF1B*	0.416667	*ITGB1*	0.010482	*JUN*	0.981788	*ACTC1*	0.023966	*VHL*	0.03003
*SMAD2*	0.416667	*COL5A2*	0.010482	*TREX1*	0.981457	*PDGFB*	0.023942	*TGFB2*	0.030018
*GLRA1*	0.416667	*ERCC8*	0.010458	*MIR210*	0.981457	*EZH2*	0.023942	*MAN2B1*	0.030005
*MAPT*	0.416667	*STAT5A*	0.010458	*TLR3*	0.981291	*IL5*	0.023936	*L1CAM*	0.02993
*TH*	0.416667	*NR3C1*	0.010452	*APP*	0.981291	*VCAM1*	0.023931	*NPC1*	0.029886
*ACTA1*	0.416667	*ALMS1*	0.010426	*IL2RB*	0.981291	*G6PD*	0.023927	*APOA1*	0.029832
*BSCL2*	0.416667	*ENG*	0.010421	*INSR*	0.981126	*PTCH1*	0.023924	*PRKAG2*	0.029803
*DSP*	0.416667	*CD79A*	0.010394	*ENO2*	0.981126	*AARS1*	0.023876	*GALC*	0.029762
*AARS1*	0.416667	*TTR*	0.010367	*RIGI*	0.981126	*LAMA2*	0.023876	*PTGS2*	0.029703
*SOX10*	0.416667	*ADA2*	0.010358	*C9orf72*	0.98096	*ATP7A*	0.023875	*ADA*	0.029702
*SMARCB1*	0.416667	*HLA-DRB1*	0.01033	*CASP8*	0.98063	*IDUA*	0.023875	*NFKB1*	0.029577
*CSF1R*	0.416667	*MAP2K1*	0.010319	*IL15*	0.98063	*ACAN*	0.023875	*LAMA2*	0.029471
*FLNC*	0.416667	*TSC2*	0.010314	*TARDBP*	0.98063	*ARSB*	0.023875	*STAT1*	0.029434
*TGFB3*	0.416667	*NEFL*	0.010308	*GNE*	0.98063	*GNPTAB*	0.023875	*IDUA*	0.02943
*AIFM1*	0.416667	*NFKB1*	0.010292	*ADAR*	0.98063	*IDS*	0.023875	*COL7A1*	0.029424
*FKTN*	0.416667	*SUMF1*	0.010258	*TBP*	0.98063	*GUSB*	0.023875	*SMPD1*	0.029358
*LAMA2*	0.416667	*BAX*	0.010248	*TBK1*	0.98063	*COL9A3*	0.023875	*SOX10*	0.029261
*ACTC1*	0.416667	*NRAS*	0.010197	*GJC2*	0.98063	*SDHB*	0.023875	*WFS1*	0.029232
*FBLN5*	0.416667	*APP*	0.010145	*IFNB1*	0.9803	*NEU1*	0.023875	*MPZ*	0.029213
*COL4A1*	0.416667	*BGN*	0.010144	*RELA*	0.9803	*FUCA1*	0.023875	*DSP*	0.029187
*ENPP1*	0.416667	*SST*	0.010106	*SELE*	0.9803	*HGSNAT*	0.023875	*DNMT1*	0.029157
*ERCC4*	0.416667	*SAMHD1*	0.010072	*CD28*	0.9803	*MAN2B1*	0.023875	*SMARCB1*	0.029155
*GALNS*	0.416667	*REN*	0.010037	*PECAM1*	0.9803	*FLNB*	0.023875	*GUSB*	0.029106
*BGLAP*	0.416667	*NKX2-5*	0.010037	*KRAS*	0.980135	*COL4A1*	0.023848	*FMR1*	0.029093
*CREBBP*	0.416667	*FKRP*	0.010031	*MAPK8*	0.980135	*COL3A1*	0.02383	*RUNX1*	0.029091
*SHH*	0.416667	*GDNF*	0.010024	*SYP*	0.980135	*LOX*	0.02383	*TNFRSF1A*	0.02908
*CASP3*	0.416667	*AKT2*	0.010015	*TAP2*	0.980135	*MYH11*	0.02383	*IDH1*	0.029064
*FGF2*	0.416667	*SOD2-OT1*	0.009964	*DPP4*	0.980135	*TNXB*	0.02383	*SLC26A2*	0.028997
*RUNX2*	0.416667	*ADRB2*	0.009964	*PAX1*	0.980135	*FN1*	0.023817	*MYH3*	0.02899
*SLC26A2*	0.416667	*SNCA*	0.009958	*GBA1*	0.97997	*NOS2*	0.023796	*FKTN*	0.028956
*MYH3*	0.416667	*SLC2A1*	0.009958	*FH*	0.97997	*SERPINC1*	0.023778	*MPO*	0.028944
*NF2*	0.416667	*KCNQ2*	0.009958	*CYCS*	0.97997	*NR3C1*	0.023776	*ENG*	0.028861
*PDGFRB*	0.416667	*DYSF*	0.009958	*F9*	0.97997	*PDGFRB*	0.023762	*CACNA1A*	0.028748
*CYP27A1*	0.416667	*LMNB1*	0.009958	*FGA*	0.97997	*ABL1*	0.023762	*KMT2D*	0.028688
*BMP2*	0.416667	*CASP8*	0.00992	*PPARGC1A*	0.97997	*GCH1*	0.023761	*MEFV*	0.028662
*EZH2*	0.416667	*IGF1R*	0.009917	*VIM*	0.97997	*AR*	0.023712	*COL4A1*	0.028636
*ENG*	0.416667	*GATA1*	0.009917	*ADAMTS13*	0.97997	*SOX2*	0.023712	*IL1A*	0.028603
*RAF1*	0.416667	*ITGB3*	0.009917	*EIF2AK2*	0.97997	*NTRK2*	0.023712	*CC2D2A*	0.028569
*PTCH1*	0.416667	*CLCN5*	0.009917	*POLR3B*	0.97997	*PLA2G6*	0.02371	*CCL2*	0.028563
*SCN11A*	0.375	*SLC29A3*	0.009917	*HSP90AA1*	0.97997	*CYP19A1*	0.02371	*MSH6*	0.028494
*IL23R*	0.375	*TNFRSF11B*	0.009902	*AGL*	0.97997	*ITGB1*	0.02371	*COMP*	0.028486
*MVK*	0.375	*IL2RB*	0.009885	*F10*	0.97997	*COL5A2*	0.02371	*INPP5E*	0.028485
*FOXP3*	0.375	*TLR3*	0.009876	*ELOVL4*	0.97997	*MMP3*	0.023643	*PRNP*	0.028452
*PMP22*	0.375	*GATA4*	0.009867	*TG*	0.97997	*BGLAP*	0.023638	*ALPL*	0.028447
*MIR146A*	0.375	*PDGFB*	0.00984	*PEX1*	0.97997	*EPO*	0.023628	*PAX6*	0.028338
*ALK*	0.375	*COMT*	0.009804	*CDH23*	0.97997	*TRPV1*	0.023627	*MKS1*	0.028174
*CALCA*	0.375	*COL4A1*	0.009792	*RNASEH2B*	0.97997	*SMPD1*	0.0236	*IKBKG*	0.028166
*MIF*	0.375	*KNG1*	0.009788	*CTSB*	0.97997	*ALK*	0.023597	*CASP8*	0.028098
*LTA*	0.375	*VEGFA*	0.009788	*COL7A1*	0.97997	*KIT*	0.023597	*NSD1*	0.028087
*CASP8*	0.375	*JUN*	0.009788	*THBS1*	0.97997	*BTK*	0.023597	*DYNC1H1*	0.028051
*KIT*	0.375	*TREX1*	0.009734	*RNASEH2A*	0.97997	*APC*	0.023597	*NR3C1*	0.028016
*PIK3CG*	0.375	*VDR*	0.009702	*GP1BA*	0.97997	*CALCA*	0.023577	*CP*	0.027999
*MALAT1*	0.375	*HBB*	0.009702	*S100B*	0.97997	*MIR34A*	0.023577	*CD36*	0.027988
*ABCA1*	0.375	*SLC26A2*	0.009683	*RNASEH2C*	0.97997	*SAMHD1*	0.023559	*SMC1A*	0.02798
*APP*	0.375	*SPP1*	0.009635	*PIGL*	0.97997	*ERBB2*	0.023532	*MEG3*	0.027908
*MIR34A*	0.375	*DST*	0.009625	*JAG1*	0.979146	*IGF1R*	0.02353	*DHCR7*	0.027844
*NR3C1*	0.375	*SYNJ1*	0.009625	*H19*	0.977338	*GATA1*	0.02353	*JAK2*	0.02784
*F5*	0.375	*HMGCR*	0.009625	*MEG3*	0.977174	*ITGB3*	0.02353	*DNMT3A*	0.027816
*BTK*	0.375	*STIM1*	0.009625	*IL1RN*	0.97701	*CLCN5*	0.02353	*BSCL2*	0.027794
*SPTLC1*	0.375	*C3*	0.009625	*CERNA3*	0.97619	*SLC29A3*	0.02353	*CHD7*	0.027747
*PLA2G6*	0.375	*IL15*	0.009607	*CSF3*	0.97619	*IL1R1*	0.023529	*EDN1*	0.027689
*SERPINA1*	0.375	*TUBB3*	0.009571	*MYH7*	0.97619	*SELENON*	0.023522	*MRE11*	0.02755
*ADIPOQ*	0.375	*AGT*	0.009561	*CCL2*	0.976027	*IGF2*	0.023508	*ABL1*	0.027544
*HIF1A*	0.375	*SERPINE1*	0.009561	*CDH1*	0.976027	*ABCC6*	0.0235	*AARS1*	0.027463
*MIR221*	0.375	*PECAM1*	0.009469	*HRAS*	0.975863	*DKC1*	0.023488	*FH*	0.027442
*MIR140*	0.375	*MIR210*	0.009452	*MTOR*	0.975863	*PIK3CG*	0.023486	*CYP19A1*	0.02743
*WAS*	0.375	*RET*	0.009449	*NRAS*	0.975863	*BMP6*	0.023486	*NEU1*	0.027427
*ERCC2*	0.375	*ACTB*	0.009427	*FKRP*	0.9757	*ABCC9*	0.023478	*ATP6V0A2*	0.027416
*SCN5A*	0.375	*INSR*	0.009396	*JAK2*	0.975536	*FKTN*	0.023472	*IDS*	0.027415
*ASAH1*	0.375	*ENO2*	0.009396	*TSC2*	0.975536	*RAF1*	0.023461	*ALDH18A1*	0.027317
*ITGAM*	0.375	*RIGI*	0.009396	*CFTR*	0.975373	*PTH*	0.023458	*GNE*	0.02725
*MSH2*	0.375	*FN1*	0.009366	*MIR125A*	0.975373	*BMP2*	0.023453	*FGF8*	0.027209
*APC*	0.375	*TBX1*	0.009352	*MAP2K1*	0.975373	*ABCB1*	0.023451	*DNM2*	0.027201
*THBD*	0.375	*ACVRL1*	0.009352	*ACTB*	0.975373	*ADA2*	0.023447	*CTSK*	0.027182
*MLH1*	0.375	*PAX1*	0.009328	*TRPV4*	0.975209	*ENG*	0.023368	*APOB*	0.027119
*WNK1*	0.375	*LTBP4*	0.009316	*MPZ*	0.975209	*ASAH1*	0.023358	*B3GAT3*	0.027034
*G6PD*	0.375	*MAPK8*	0.009297	*TNFRSF11B*	0.975209	*GH1*	0.023328	*EGF*	0.026997
*SAMHD1*	0.375	*TAP2*	0.009297	*ALMS1*	0.975209	*CASR*	0.023328	*TARDBP*	0.02696
*BMP6*	0.375	*DPP4*	0.009297	*RET*	0.975046	*CST3*	0.023328	*TPM2*	0.026952
*CP*	0.375	*PDGFRB*	0.009292	*MECP2*	0.97472	*IGFBP3*	0.023328	*HNF1B*	0.026941
*ADA2*	0.375	*DKC1*	0.00928	*FIG4*	0.97472	*MAF*	0.023328	*PIK3CD*	0.026905
*GPHN*	0.375	*CD28*	0.009274	*ATM*	0.974556	*IDH2*	0.023328	*MYH11*	0.0269
*KIF1A*	0.375	*IFNB1*	0.009265	*FGFR1*	0.974556	*CTSD*	0.023273	*TREX1*	0.026889
*NEFL*	0.375	*RELA*	0.009265	*NFKB1*	0.974393	*FLNC*	0.023221	*SYNE1*	0.026868
*ABCC9*	0.375	*SELE*	0.009265	*MAPT*	0.974393	*GATA4*	0.023187	*PLA2G6*	0.026839
*REN*	0.375	*TARDBP*	0.00926	*COL4A1*	0.974393	*SST*	0.023178	*ADA2*	0.026823
*EPO*	0.375	*GNE*	0.00926	*GALNS*	0.974393	*TPM3*	0.023172	*MVK*	0.026807
*SPTAN1*	0.375	*ADAR*	0.00926	*VDR*	0.97423	*FMR1*	0.023169	*JUN*	0.026798
*SELENON*	0.375	*TBP*	0.00926	*HBB*	0.97423	*MYH14*	0.023163	*AIFM1*	0.026733
*HSPB1*	0.375	*TBK1*	0.00926	*TUBB3*	0.97423	*DNMT3A*	0.023162	*SCN5A*	0.026706
*GAA*	0.375	*GJC2*	0.00926	*ENG*	0.97423	*LRP5*	0.023162	*ICAM1*	0.026684
*RB1*	0.375	*HIF1A*	0.009248	*REN*	0.974067	*SDHC*	0.023162	*GALNS*	0.026653
*MYH14*	0.375	*EPHX1*	0.009239	*AIFM1*	0.974067	*IHH*	0.023162	*COL5A2*	0.026649
*VHL*	0.375	*NGF*	0.009231	*NKX2-5*	0.974067	*SPARC*	0.023162	*GATA4*	0.026592
*NKX2-5*	0.375	*ACTA2*	0.009222	*TTR*	0.973904	*EBP*	0.023162	*FBLN5*	0.026574
*DYNC1H1*	0.375	*SYP*	0.009219	*FN1*	0.973904	*WNT1*	0.023162	*ERCC4*	0.026544
*GDAP1*	0.375	*TPM2*	0.009208	*CTSK*	0.973741	*MMP14*	0.023162	*CTSD*	0.026509
*IGF2*	0.375	*FIG4*	0.009146	*GATA4*	0.973741	*SGSH*	0.023132	*FN1*	0.026505
*SLC17A5*	0.375	*MECP2*	0.00914	*AARS1*	0.973579	*GDNF*	0.023123	*ACAN*	0.026489
*TPM3*	0.375	*TSC1*	0.009103	*LAMA2*	0.973579	*SPTAN1*	0.023113	*CACNA1C*	0.026448
*CTSK*	0.375	*TRAF3IP2*	0.009052	*VEGFA*	0.973416	*DYNC1H1*	0.023113	*CDKL5*	0.026434
*FN1*	0.375			*PDGFB*	0.973416				
*PTH*	0.375								
*EGR2*	0.375								
*HLA-A*	0.375								
*GNRH1*	0.375								
*ANO5*	0.375								
*CDKN1A*	0.375								
*SERPINC1*	0.375								
*COL9A2*	0.375								
*LDLR*	0.375								
*CYP19A1*	0.375								
*RUNX1*	0.375								
*GJB2*	0.375								
*COL3A1*	0.375								
*FASLG*	0.375								
*ALDH18A1*	0.375								
*GDNF*	0.375								
*POLR3A*	0.375								
*ITGB1*	0.375								
*IFNA1*	0.375								
*LOX*	0.375								
*MYH11*	0.375								
*CD79A*	0.375								
*ABCC6*	0.375								
*COL5A2*	0.375								
*TNXB*	0.375								
*TIMP1*	0.375								
*SUMF1*	0.375								
*MBTPS2*	0.375								
*BGN*	0.375								
*PIK3C2A*	0.375								
*TBX4*	0.375								
*RMRP*	0.375								
*DVL1*	0.375								
*ATP7A*	0.375								
*UBA1*	0.375								
*AR*	0.375								
*IDUA*	0.375								
*ACAN*	0.375								
*ARSB*	0.375								
*GNPTAB*	0.375								
*IDS*	0.375								
*GUSB*	0.375								
*FMR1*	0.375								
*SMPD1*	0.375								
*CTSD*	0.375								
*COL9A3*	0.375								
*SDHB*	0.375								
*NEU1*	0.375								
*FUCA1*	0.375								
*HGSNAT*	0.375								
*SGSH*	0.375								
*TSC1*	0.375								
*MAN2B1*	0.375								
*SOX2*	0.375								
*FLNB*	0.375								
*NTRK2*	0.375								
*ACTA2*	0.375								
*GATA4*	0.375								
*ABL1*	0.375								
*NOTCH2*	0.375								
*DKC1*	0.375								

## Data Availability

The original contributions presented in this study are included in the article and Supplementary Materials. Further inquiries can be directed to the corresponding authors.

## Supplementary Materials

The following supporting information can be downloaded at: https://www.mdpi.com/article/10.3390/cimb48020199/s1, Figure S1. Degree centrality analysis of Parkinson’s symptom–gene network and top 5% gene list. Figure S2. Betweenness centrality analysis with network visualization and top 5% gene list. Figure S3. Closeness centrality results highlighting top-ranked genes in the network. Figure S4. Eigenvector centrality analysis showing key influential genes and top 5% gene list.
