# Supplementary figures and images for: Therapeutic Insights and Immune Pathway Connections Revealed by Core Symptom Gene Network Analysis in Ankylosing Spondylitis
Source: Curr Issues Mol Biol. 2026 Feb 11;48(2):199. doi: 10.3390/cimb48020199 (PMC12939522; doi:10.3390/cimb48020199)

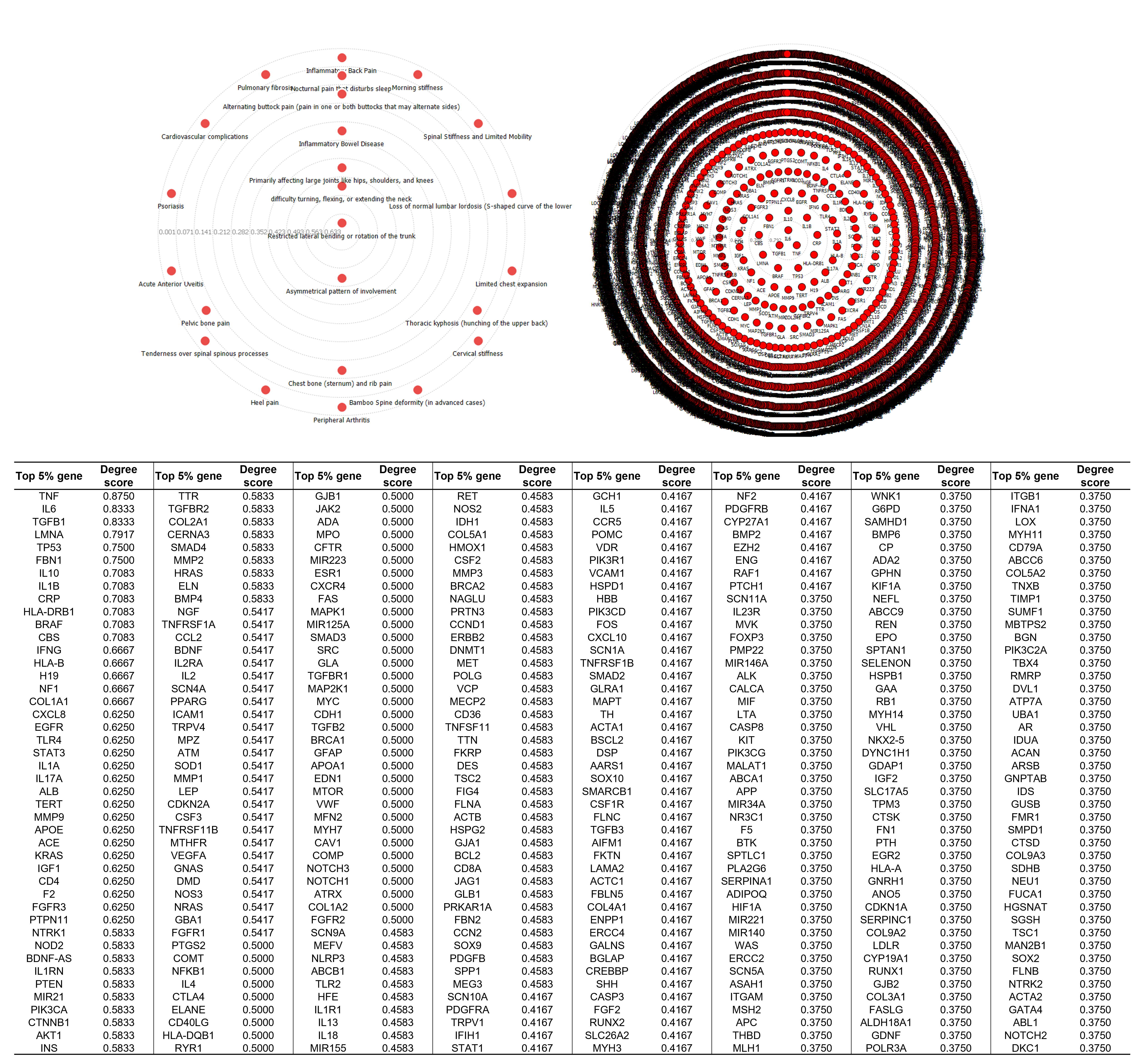

Supplement: Supplementary file 1 [file cimb-48-00199-s001.zip › Fig.S1.tif]

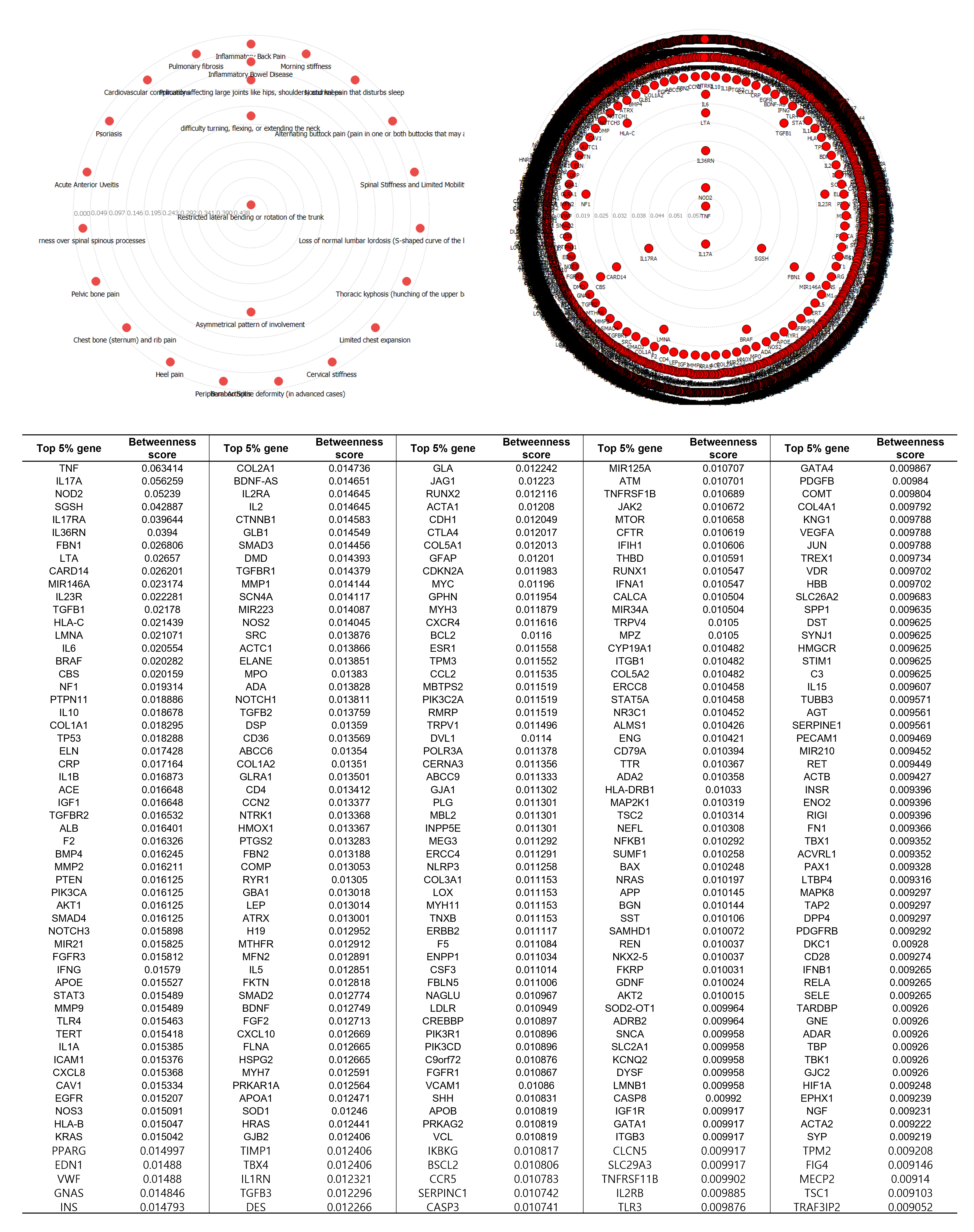

Supplement: Supplementary file 1 [file cimb-48-00199-s001.zip › Fig.S2.tif]

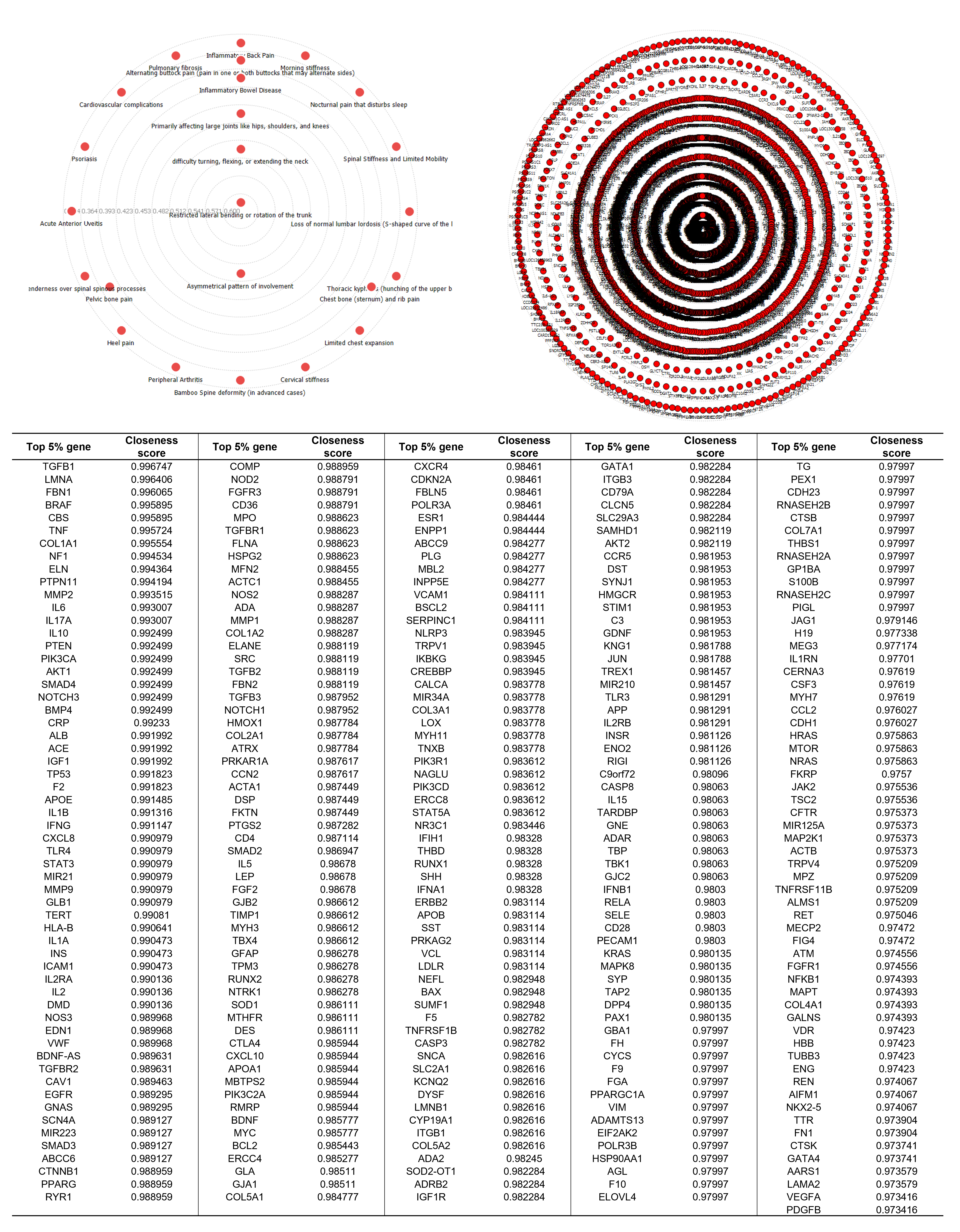

Supplement: Supplementary file 1 [file cimb-48-00199-s001.zip › Fig.S3.tif]

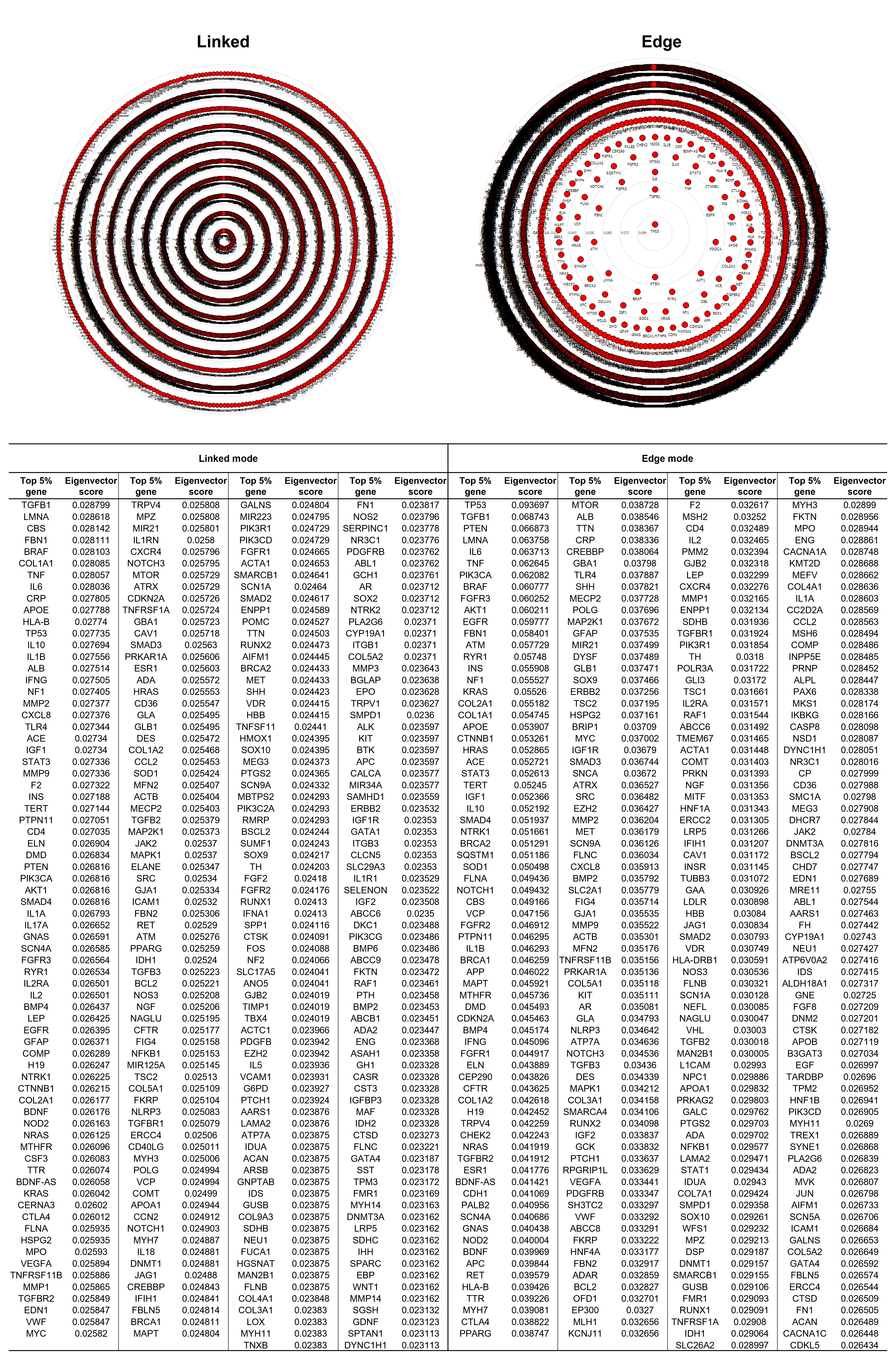

Supplement: Supplementary file 1 [file cimb-48-00199-s001.zip › Fig.S4.tif]
